# Implementing a mentoring program for clinical research professionals: A novel professional development initiative for university health research staff

**DOI:** 10.1017/cts.2023.655

**Published:** 2023-10-31

**Authors:** Elias Samuels, Ellen Champagne, Angela K. Lyden, Gloria J. Harrington, Reema Kadri, Jennifer A. Miner, Sana Shaikh, Phillip A. Ianni, Brenda Eakin, Susan Murphy

**Affiliations:** 1 Michigan Institute for Clinical and Health Research, University of Michigan, Ann Arbor, MI, USA; 2 Office of Research, Michigan Medicine, Ann Arbor, MI, USA; 3 Department of Psychiatry, Michigan Medicine, Ann Arbor, MI, USA; 4 Department of Family Medicine, Michigan Medicine, Ann Arbor, MI, USA; 5 Department of Physical Medicine and Rehabilitation, University of Michigan, Ann Arbor, MI, USA; 6 Department of Anesthesiology, University of Michigan Michigan Medicine, Ann Arbor, MI, USA

**Keywords:** Mentoring, clinical research professionals, professional development, implementation, program evaluation

## Abstract

Clinical and translational research relies on a well-trained workforce, but mentorship programs designed expressly for this workforce are lacking. This paper presents the development of a mentoring program for research staff and identifies key programmatic outcomes. Research staff participating in this program were matched with a senior mentor. Focus groups were conducted to identify key program outcomes. Surveys were administered throughout the program period to assess participants’ experience, gains in skill, and subsequent careers. Analysis of the resultant qualitative and quantitative data are used to characterize the implementation and impact of the program. A total of 47 mentees and 30 mentors participated in program between 2018 and 2023. A comprehensive logic model of short-, intermediate- and long-term outcomes was developed. Participants reported positive valuations of every programmatic outcome assessed including their program experience, learning and research careers. The pool of available mentors also grew as new mentors were successfully recruited for each cohort. This mentorship program developed and implemented by senior research staff successfully provided junior research staff with professional development support, mentorship, and professional development opportunities. Junior and senior health research staff built mentoring relationships that advanced their clinical and translational research careers.

## Introduction

Clinical and translational research relies on a workforce of highly skilled professionals who have the understanding and resources needed to carry out the tasks required to advance translational research [1]. Evidence suggests that mentorship improves the training and the performance of these research team members [2–3]. This paper shows how the development of a mentoring program for this workforce contributes to participants’ pursuit of their career goals.

This paper concerns a mentoring program for clinical research professionals (CRP) at the University of Michigan (U-M). CRP is an umbrella term that comprises various job titles, including for example clinical research coordinators, research nurses, and clinical research associates [4–6]. CRPs are responsible for performing a variety of tasks, including but not limited to participant recruitment, monitoring studies, data collection, and the development of protocol documents. However, opportunities for mentorship by senior CRPs are often lacking, and career advancement pathways are not always clear [7–8]. The participants in the program presented here are pursuing research careers as staff who have not been appointed to any faculty, research, or clinical track at the U-M. The definition of “research careers” used for this paper was adapted from the definition developed by the NIH’s National Center for Advancing Clinical and Translational Science (NCATS) [9].

## Background

The U-M Medical School (Michigan Medicine) has previously supported initiatives for mentoring the health research workforce in ways that have been shown to legitimize and professionalize their research careers [10]. In 2016, the Michigan Medicine Clinical Trials Support Office (CTSO) was created to be a central hub that provides enterprise-wide leadership, standards, policies, and a common infrastructure [11]. In 2018, Michigan Medicine faculty and staff in the Behavior, Function, and Pain unit of the CTSO and in the Department of Psychiatry developed a mentorship program called the Staff Enrichment Program for research professionals (STEP.up) designed for clinical and translational research staff working in any U-M college, school, or research center.

Michigan Medicine’s CTSO and the Michigan Institute for Clinical and Health Research (MICHR) partnered to provide financial and administrative support for the STEP.up program. This partnership was supported through NCATS and its Clinical and Translational Science Awards (CTSA) program [12]. The CTSA program provides institutions like Michigan Medicine with the capacity needed to support multistage scientific investigations that accelerate the transformation of fundamental medical discoveries into new opportunities to improve the health of individuals and their communities [13]. STEP.up was designed to complement existing mentorship programs available to faculty through Michigan Medicine, U-M, and other organizations that serve Principal Investigators [14–17].

### Program Design

The STEP.up program is administered by two senior clinical research professionals who developed, implemented, and are maintaining the program. An Advisory Board with rotating membership guides these lead administrators’ work, facilitates the application and mentor matching process, and provides continuous oversight of the program’s operations and evaluation. This board consists of several senior clinical and health research staff. The board meets quarterly throughout the year, supports program operations, monitors the development of the mentoring relationships, and evaluates the impact of the program.

Cohorts of mentees are admitted to the program annually, with the number of new mentees ranging from 6 to 12. Mentors are recruited each year, thereby growing the pool of trained mentors over time. The application period is advertised through regular communications disseminated by the CTSO, MICHR, and other U-M schools and departments. Applicants are required to provide a letter of support from their supervisor or Principal Investigator that confirms the applicant will be able to dedicate the time required for program activities and mentor meetings. All applicants must confirm that they have had formal training in the ethical conduct of research.

Mentee and mentor applications go through an initial review by the program co-leads. Personas of a mentor and mentee were developed to inform this process [18]. This initial review is to evaluate compatibility with the established mentor and mentee personas and is intentionally inclusive. Review assignments are then created for the Advisory Board. Board members do not review candidates within their own reporting chain or department/unit. Each mentee application is randomly assigned three independent reviewers and is scored on a scale of one to five, with 1 representing “outstanding” and 5 being “unable to judge,” across five domains: (1) motivation for formal mentoring, (2) articulated professional development goals, (3) goals align with program objective and structure, (4) interest in maintaining connection to research regardless of career aspirations, and (5) overall impression of prospective mentee as candidate for program. Reviewers are encouraged to suggest matches based on a mentor’s experience and a mentee’s articulated career goals, background, and interests.

Applications are sorted from best score (i.e., best scores are those lowest in numeric value) to worst and the board is convened to discuss mentee and mentor pairings. Program co-leads facilitate the discussions around acceptance and mentor pairings. The group starts with the third who scores the best, then moves to the third who scores the worst and either determines that they do not meet criteria for the program or may try to match with a suitable mentor. The group then moves through the remainder of the mentees on the list to try to identify suitable pairings. The discussion is centered on suitability for the program at the mentee’s current career stage, career aspirations, desire to stay in research, and whether there is a potential mentor match available. The selected mentor must be from an academic department different from the mentee’s. This allows for a greater degree of confidentiality between mentor/mentee pairs. Matching mentees with mentors in other departments also promotes exposure to broader sets of professional experiences and scientific areas of study.

### Program Implementation

The program is structured into three phases spanning a year. At the start of the year, the Advisory Board meets to evaluate applications and admits participants to the program. Each cohort of mentees attends a two-hour orientation where they receive information about the structure and goals of the program. Participants also receive training about mentor-mentee relationships, based on training developed by the Center for the Improvement of Mentored Experiences in Research (CIMER) [19]. Program mentors attend a separate two-hour training session based on modules created by CIMER, including Maintaining Effective Communication, Aligning Expectations, Promoting Professional Development, Addressing Equity and Inclusion, and Articulating your Mentoring Philosophy and Plan. These orientation sessions are led by individuals who received mentorship training endorsed by the National Research Mentoring Network [20].

A minimum of one hour per month of required contact between mentors and their mentees provides time to develop the mentoring relationship, although the actual regularity or duration of this contact varies. The first of these meetings is dedicated to setting and discussing career goals. Mentee/mentor pairs were also used to complete questionnaires. These questionnaires include questions about the progress being made toward identified goals. As outlined in the Methods section, these questionnaires were administered anonymously during the first two years of the program and then transitioned into personalized surveys to enable responses to be tracked.

Professional development and networking, and further mentor training opportunities are offered to participants during the program. Key mentor responsibilities, roles, and expectations are discussed, and the importance of confidentiality and following ethical guidelines is emphasized throughout this training. A concluding ceremony is held at the end of the eight-month mentoring period to present participants with certificates recognizing their progress, typically delivered by their mentors. The final two months of the year are used to conduct program evaluations with the participants, convene the Advisory Board to review results, and adjust programing for future cohorts.

## Methods

The program was evaluated through a partnership with the Center for Education Design, Evaluation and Research at the U-M School of Education [21]. The development and evaluation of this mentoring program were reviewed and approved by the University of Michigan Institutional Review Board for Health and Behavioral Sciences (HUM00149662) in 2018. The outcomes of the STEP.up program were identified and evaluated using an exploratory mixed methods design [22].

Focus groups with mentors and other mentees were conducted virtually at the end of the program in 2019 with participants recruited from the first two cohorts. The 1-hour focus groups were conducted with every participant who volunteered to participate. The small size of this sample (*N* = 10) is a key limitation as detailed in the Discussion section.

These focus groups were conducted with the purpose of identifying the ways that the program affected mentees’ professional development over time. These focus groups were recorded and transcribed. The transcriptions were coded using Dedoose Version 9.0.17 [23], and analyzed using Grounded Theory [24]. The results were then presented to the Advisory Board.

These results were used by the STEP.up administrators and Advisory Board to identify programmatic outcomes during a series of meetings conducted throughout 2019 and 2020. Short-, intermediate-, and long-term outcomes were defined by the Advisory Board using logic models [25]. These outcomes were categorized into four conceptual levels that included participants,’ (1) Learning, (2) Experience, (3) Behavior, and (4) programmatic Results [26].

Surveys were used to collect feedback related to participants’ learning and experience. Pre-program surveys were sent to mentees in advance of orientation sessions to collect feedback regarding their professional goals, interests, and mentoring and research skills. Feedback surveys adapted from the Mentorship Profile Questionnaire were also administered to mentees and mentors receiving mentoring training [27]. Surveys were also used to track mentee’s relevant post-program behaviors, specifically regarding the progress made toward professional goals and ongoing mentoring activity.

Secondary data collection was conducted annually to track participants’ health research careers. Key participant information and outcomes are collected and managed using REDCap [28]. These data were also used by the Advisory Board to review programmatic impact.

## Results

Since STEP.up was launched in 2018 five cohorts have been enrolled (Table 1). The pool of active mentors increased steadily throughout the duration of the program. The professional and demographic characteristics of the program participants are summarized in Table 2. On average, the mentees had over two years of research experience at U-M (*N* = 47, Mean = 2.8, SD = 2.4) and over 4 years of total research experience (*N* = 47, Mean = 4.7, SD = 3.8). Changes in the collection of professional and demographic characteristics of the program participants were made in 2021. Before 2021, only participants’ job titles, funding sources, and years of research experience were collected. After 2021, participants’ sex, race, ethnicity, and disability status were collected; participants were also asked if they viewed themselves as being underrepresented minorities in the extramural research workforce after being shown a definition of the term [29]. The inconsistency of data collection on participants’ background is another key limitation, as emphasized in the Discussion.

**Table 1.** STEP.up mentee and mentor cohorts and applications

Years	Cohort	Number of applications received	Number of mentees	Number of new/total mentors
2018–2019	1	9	8	8/8
2019–2020	2	17	12	5/10
2020–2021	3	13	6	4/7
2021–2022	4	14	10	7/10
2022–2023	5	14	11	6/10
Totals		67	47	30

**Table 2.** STEP.up mentee professional and demographic characteristics

Background characteristics	*N*	%
**Job titles**		
Coordinators	31	66%
Research Area Specialists	5	11%
Research Assistants	6	13%
Lab Managers	3	6%
Data Managers	1	2%
Research Analysts	1	2%
Job Titles Total	47	100%
**Funding support by type**		
Federal	43	48%
FDA/Industry	10	11%
Small budget/Internal Funds	19	21%
Foundations	9	10%
Other	9	10%
Funding support total	90	100%
**Sex**		
Female	18	86%
Male	3	14%
Sex total	21	100%
**Race**		
White	17	81%
Black or African American	2	10%
Asian	1	5%
Choose not to answer	1	5%
Race Total	21	100%
**Ethnicity**		
Hispanic (regardless of race)	2	10%
Non-Hispanic	18	86%
Choose not to answer	1	5%
Ethnicity total	21	100%
**Disability**		
Has a disability	1	5%
Has no disability	20	95%
Disability total	21	100%
**Underrepresented minorities in extramural research**		
Underrepresented minority	7	33%
Not an underrepresented minority	14	67%
Underrepresented minority total	21	100%

### Qualitative Results

Two focus groups, one with five mentees and one with five mentors, yielded evidence of how the program could be impactful. These focus groups were intended to define the potential outputs and outcomes of the program, not to inform generalizations about the magnitude of the impact of the program. The results of these focus groups were used solely to characterize the ways in which the program could affect the participants using logic models.

The focus group participants had varied reasons for joining STEP.up. Some mentees were looking for professional development and saw it as an opportunity for career growth. Others stated that the program was recommended by their supervisor. Some mentors wanted to participate as a professional service, to give back by helping others have more career opportunities. Other mentors expressed wanting to connect with more colleagues working across departments and disciplines. Some said that they enjoyed the mentoring experience which was difficult to obtain otherwise.

Mentees indicated the program contributed to their personal growth, helped them meet contacts in their field, and improved their communication skills. Some mentees said that their mentor served as a sounding board and that they could trust their mentor to speak confidentially about job-related issues. Mentees also felt the mentoring program could help them set short- and long-term career goals and become more confident in their abilities. According to one mentee, “Using my mentor’s advice, I am gradually learning skills I wanted to learn for professional development.” Another noted, “I have really enjoyed meetings with my mentor. Our meetings have led me to think about ways to expand my research skills that weren't on my radar in the first place.”

Mentors thought the program benefitted both the mentees and mentors. They stated that interactions with mentees taught them about new areas of health research and scientific methods. The mentors also said they became better managers because of the program, and more creative and confident in themselves as professionals. They described how the program enabled them to meet other mentors, and that the program showed mentees fitting career paths in health research. One stated,

“[STEP.up] definitely helped me learn how to figure out how to be the type of mentor I wanted to be. I think both my mentee and I were able to set specific goals for our jobs, she actually was able to get a promotion and new job title during this time (I'm still working on mine). I also found the STEP.up program to be a wealth of knowledge for research staff, and I plan to keep tapping into it as I move along my career.”

While mentees and mentors expressed that they benefited from the program, mentors also reported encountering distinct challenges in forming effective mentorship relationships. While mentees were very positive about the experience with their mentors in STEP.up, mentors described several challenges related to learning about their mentee’s professional goals. Mentors reported that their mentees initially didn't have a goal in mind, often weren't focused sufficiently on their own professional development, and were occasionally unfamiliar with the purpose of mentoring. Mentees said the greatest potential impact of the program regarded the ways that it might have helped them get off their “research island” and give them an opportunity to talk to others with a different set of experiences. For mentors, the most potentially beneficial aspects of the program regard its potential to validate the staff role and formalize the mentee-mentor relationship.

**Figure 1.**
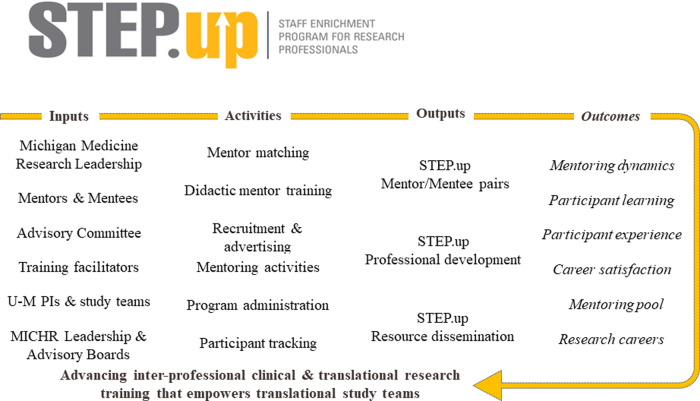
STEP.up logic model.

These results were used to identify and define the programmatic inputs, activities, outputs, and outcomes shown in the logic model developed by the study team (Fig. 1). Results of quantitative analyses of other short-, intermediate-, and long-term outcomes are presented below. Notably, the focus groups enabled mentees and mentors to provide recommendations for potential improvements to the program’s design and administration. Mentees provided several suggestions, including helping mentees be better prepared for their mentor meetings, having more structured discussion topics provided for meetings, doing more activities as a group, dedicating more time to talk with other mentees, and having a slower-paced introductory meeting. Mentors’ recommendations included having more structured check-ins with other mentors, getting more input on how to use professional development resources, and using meetings to focus on networking with mentees and mentors. These recommendations were provided to the STEP.up administrators and Advisory Board.

### Quantitative Results

Surveys adapted from the Mentorship Profile Questionnaire were administered to evaluate the mentoring dynamics of the participants receiving mentor training every year (Table 3). Pre-program surveys were sent to mentees before orientation sessions to collect feedback regarding their professional goals, interests, and mentoring skills. To assess learning participants were asked to self-assess their skills on the key learning outcomes shown in Table 3. Mentees’ lowest average self-rating was for setting life goals (Mean = 4.3), and their highest average self-rating (Mean = 6) was given for multiple skills in Active Listening, establishing a relationship based on trust, considering how personal and professional differences may impact expectations, and working effectively with mentors whose personal background is different.

**Table 3.** STEP.up program outcomes

Outcome type	Survey question	Training type	N	Mean	SD
Participant learning	Identify strategies to help develop my mentoring relationships in situations where the initial connection doesn't feel as strong	Mentor training	15	4.2	0.08
Participant learning	Define boundaries for my relationships with my mentee	Mentor training	15	4.5	0.6
Participant learning	Articulate philosophical or behavioral differences between my mentor and managerial roles	Mentor training	15	4.2	0.9
Participant learning	Recognize differences in communication styles and identify strategies for communicating across styles	Kickoff Mentee training	41	4.2	0.9
Participant learning	Identify strategies for recognizing and addressing barriers to communication	Kickoff Mentee training	40	4.0	0.9
Participant experience	I would recommend this training to my colleague	Mentor training	29	4.4	0.9
Participant experience	Helped me describe my initial STEPs in preparing for my initial meeting with my mentee	Mentor training	15	4.2	0.9
Participant experience	Attending today’s session was a valuable use of my time	Kickoff Mentee training	41	4.5	0.6
Participant experience	Learned skills and strategies through this session that I can use in my professional relationships	Kickoff Mentee training	41	4.0	0.8
Participant experience	The session was helpful for sharing useful information on how to embark on your mentor-mentee relationship	Kickoff Mentee training	41	4.3	0.6

The measures used to assess participants’ experience in the program are also shown in Table 3. The participants indicated that the facilitator(s) were very effective or effective (55 of 56 participating mentees and mentors, 98%) in guiding the discussion, and very effective or effective (53 of 57, 93%) at, “sharing useful information on how to embark on the mentor-mentee relationship.” Most of the participants’ qualitative responses in these surveys indicated that the mentor-mentee relationship was effective and productive.

The long-term outcomes of the program were measured using two metrics. One metric was adapted from the definition of a “Research Career” developed by NCATS through their Common Metrics Initiative [12]. The vast majority (96%) of the 47 participants in the first three cohorts confirmed through follow-up surveys administered at the end of the program that they were pursuing a health research career. Publicly available information about the participants was used to confirm that all these respondents (100%) were currently in a Research Career at the time of this study. One individual in the second cohort of the program left the institution and could not be contacted and was lost to follow-up; this is another key limitation described in the Discussion. The other metric used to assess the impact, or result, of the program was the growth of the mentoring pool for the program, which grew each year (Table 1).

## Discussion

There are few mentoring programs for clinical research professionals. This study provides evidence suggesting that STEP.up had a positive and lasting impact on the professional development and advancement of mentees. In addition, the short-, intermediate-, and long-term outcomes identified through this study can be used to benchmark programmatic improvement of STEP.up, and of similarly structured mentoring programs.

The findings of this study suggest that key barriers and facilitators may have affected the implementation and impact of STEP.up. Key barriers regard a range of organizational, administrative, financial, and cultural factors. These include (a) a lack of time for staff to serve as mentors or mentees; (b) feelings of imposter syndrome among new mentors; and (c) limited funding to expand the number of mentees admitted in each cohort and provide participants with professional development funding or opportunities. The impact of the program may have also been facilitated by the non-binding permission and encouragement that participants received from their Principal Investigators or supervisors to dedicate considerable time to the programs’ professional development and mentoring activities.

The limitations of this study must be emphasized. This program was created without first specifying specific outcomes by which the impact of the program was to be assessed. Small sample sizes for the focus groups and low survey response rates to surveys limit the degree to which STEP.up can be inferred to have had a substantial impact on the participating mentees’ careers. The variation in response rates to the participant surveys administered during the program is also a limitation. The timing of the administration of these surveys varied across years as the structure and start-date of the program changed, particularly following the onset of the COVID-19 pandemic. Response rates ranged from above 95% to below 25% across the more than dozen participant surveys administered over the first five years of the program.

Other key limitations of this evaluation regard the lack of demographic diversity of the participants and possibility that those individuals who were selected into the program were research staff already predisposed and positioned to succeed in their research careers. The process by which individuals were selected to participate in the program could have contributed to the lack of greater demographic diversity in the participating cohorts. The authors recognize and support the need for more mentoring opportunities to be made available to all health research staff, particularly those who are most at risk of leaving the profession.

The results of this study demonstrate the value of organizational collaboration and partnership among disparate groups working within academic medical centers. It is likely that this program could not have been developed, implemented, maintained, and evaluated without the dedication and enthusiasm of those who created the program. Academic medical centers seeking to cultivate the professional development and advancement of their clinical and translational research workforce should endeavor to provide greater financial and organizational support for similar mentoring programs. It is reasonable to hypothesize that the provision of greater financial and organizational support would serve to increase the impact of mentoring programs designed for this workforce.

Further research should evaluate the generalizability of these findings across specialists of clinical research professionals, as well as with those with above- and below-average years of experience in their roles. Future studies should also evaluate the long-term impact of these programs on the research careers of all participating clinical research professionals using valid comparison groups. Quasiexperimental methods, such as propensity score matching, have been used to demonstrate the impact of mentored research awards on the work of clinical and translational investigators, and similar methods should be used to evaluate the impact of mentoring opportunities provided to the health research workforce that supports their scientific work [30].
